# Plasmon Induced Photocatalysts for Light-Driven Nanomotors

**DOI:** 10.3390/mi12050577

**Published:** 2021-05-19

**Authors:** Enrique Contreras, Christian Palacios, I. Brian Becerril-Castro, José M. Romo-Herrera

**Affiliations:** 1 Centro de Nanociencias y Nanotecnología, UNAM, Km 107 Carretera Tijuana-Ensenada, C.P., Ensenada 22800, BC, Mexico; cpalacios@ens.cnyn.unam.mx (C.P.); g2_becerril13@ens.cnyn.unam.mx (I.B.B.-C.); 2 Posgrado en Nanociencias, Centro de Investigación Científica y Estudios Superiores de Ensenada, Carretera Ensenada-Tijuana No. 3918, Zona Playitas, C.P., Ensenada 22860, BC, Mexico

**Keywords:** micromachines, nanomachines, LSPR, Photocatalysis

## Abstract

Micro/nanomachines (MNMs) correspond to human-made devices with motion in aqueous solutions. There are different routes for powering these devices. Light-driven MNMs are gaining increasing attention as fuel-free devices. On the other hand, Plasmonic nanoparticles (NPs) and their photocatalytic activity have shown great potential for photochemistry reactions. Here we review several photocatalyst nanosystems, with a special emphasis in Plasmon induced photocatalytic reactions, as a novel proposal to be explored by the MNMs community in order to extend the light-driven motion of MNMs harnessing the visible and near-infrared (NIR) light spectrum.

## 1. Introduction

Micro/nanomachines (MNMs) are human-made devices (colloids) with dimensions ranging between 10 nm and 10 µm, with motion through a specific direction, and can perform different tasks in aqueous solutions [1,2].

There are different routes for powering these devices, such as: (i) chemical [3,4], (ii) magnetic [5,6], (iii) ultrasound [7,8], (iv) electrical [9,10], and (v) light [11,12,13,14,15]. Among these, light-driven MNMs have gained attention due to the possibility to convert light energy into kinetic energy as “fuel-free MNM” [16,17,18]. 

To convert the photons into kinetic energy there is a common approach, which is to use an asymmetrical particle (Janus) and a semiconductor with a well-established band gap [19,20]. The major challenge for the light-driven MNMs is the source of the energy, and the penetration depth of this source of energy [16]. Therefore, it is important to reduce the energy to a lower frequency (compared to the UV) such as visible or near-infrared (NIR) light. 

In recent years, plasmonic photochemistry has gained attention due to the possibility of performing chemical reactions employing visible light and NIR light [21,22]. Thereafter, several applications have appeared such as sensors [23,24], chemical conversion [25,26], and photo-electrochemical conversion [27,28,29]. One of the main driving forces on this arrives from plasmon induced energetic carriers [30,31].

However, very little attention has been paid to harness these energetic charge carriers as a source of energy for MNMs propulsion. In this review we attempt to go beyond the current trend of photochemistry and present a perspective with the potential use of plasmon induced photocatalytic activity for light-driven micro/nanomotors. We start by describing titanium dioxide (TiO_2_) based photocatalysts as light-driven micro/nanomotors in Section 2 as the most common use of light for MNMs propulsion. Then, we introduce the physical properties of Plasmonic NPs and hot charge carriers’ generation in Section 3, as the main concepts for plasmonic photocatalysis. Afterwards, we describe some illustrative reports on plasmonic photocatalysts for light-driven reactions in Section 4; and Plasmonic NPs—TiO_2_ hybrid systems for light-driven reactions, including some examples of Janus –like hybrid systems breaking the symmetry of the assemblies for potential use in propelled motion, in Section 5; to continue with some ideas around plasmon-driven chemical reactions for propelled nanomotors in Section 6. Finally, we end the paper with a brief conclusion and future perspectives in Section 7.

## 2. TiO_2_-Based Photocatalysts as Light-Driven Micro/Nanomotors

MNMs made from photoactive semiconductors have several advantages when compared to those made with traditional metal catalysts, such as lower production costs, resistance to photocorrosion/catalyst poisoning, and controllable activity (via on/off switching of incident light) [14]. At the heart of every semiconductor-based MNM there is one crucial process: electron-hole separation. Triggered by photons of energy equal or greater than its bandgap, electrons are promoted to higher energy states in the conduction band of the semiconductor, leaving positively charged vacancies (also known as ‘‘holes’’) in its valence band. Without any external influence, most of these generated charge carriers end up returning to their ground state in a process commonly referred to as ‘‘electron-hole recombination’’. However, some of them can migrate to the surface of the semiconductor without suffering recombination events. Once there, charge carriers react with available redox species such as water or H_2_O_2_, separating them into H^+^ and O_2_, as well as superoxide (•O_2_^−^) and hydroxyl (•OH^−^) radicals, highly oxidative species which can decompose a great variety of organic compounds [12,14,32,33,34]. The rate of chemical conversion depends directly on the amount of charge carriers available at the motor surface, which can be enhanced by increasing the energy/intensity of incident light and/or reducing the chances of electron-hole recombination. The latter is often done by introducing elements that can receive and trap photogenerated electrons into the semiconductor matrix, which are mostly metals like Pt, Au, Ag, Fe, and Cu [35]. Chemical conversion at the MNM surface, and thus motion efficiency, can also be enhanced to a lesser extent via the removal of crystalline defects and increase in surface area [36].

For many years, Pt/Au-TiO_2_ Janus nanoparticles have been used for the study of semiconductor-based MNMs due to the high photocatalytic activity, great stability, and easy crystalline phase control of TiO_2_, as well as the excellent electron-trapping and catalytic properties of Pt/Au [14,19,20,32,37,38,39,40]. Although these properties are desirable to ensure high activity over extended time periods [32,33,41,42], the use of Au/Pt tends to be costly and not feasible for mass production [43]. Additionally, the conditions required to achieve optimal performance in traditional TiO_2_-based MNMs (high-intensity UV light and high H_2_O_2_ concentrations) can be harmful for many environments [44]. Such inconveniences have driven research groups to design economic alternatives for the fabrication of highly active MNMs under low-energy light and fuel-free/biocompatible fuel conditions. In the case of TiO_2_-based MNMs, it has been shown that photoactive response to low energy light can be obtained and tuned from UV to the IR region via quick annealing of TiO_2_ nanoparticles [40], photosensitizing of TiO_2_ particles with organic dyes [45] or carbon nanostructures [46], the use of organic dyes as fuel [47] and introduction of other semiconductors like Cu_2_O or ZnO to the motor structure [14,48]. The possibility of using lower bandgap semiconductors as a main component has also been explored, obtaining promising results under visible and NIR light for MNMs based on ZnO [39], Ni-modified ZnO [43], ZnO-AgBr [48], Au-ZnO [49], Pt-ZnO [36,50], Carbon dot-Cu_2_O [51], Cu_2+1_O [44], Fe_2_O_3_ [52,53,54], Fe_3_O_4_ [55], Bi_2_O_3_ [56,57], C_3_N_4_ [58], MnO_2_ [59,60], WO_3_ [61], Si [62], and Zn_X_Cd_1−X_Se-Cu_2_Se-Pt [63].

Redox reactions on the surface of semiconductors generate more product molecules/ions than those necessary to initiate them, leading to the development of local gradients in asymmetric micro/nanomotors [12,32,33,34] and enabling their propulsion via one or more of three main mechanisms: (1) diffusiophoresis, (2) electrophoresis, and (3) bubble propulsion.

**Figure 1:**
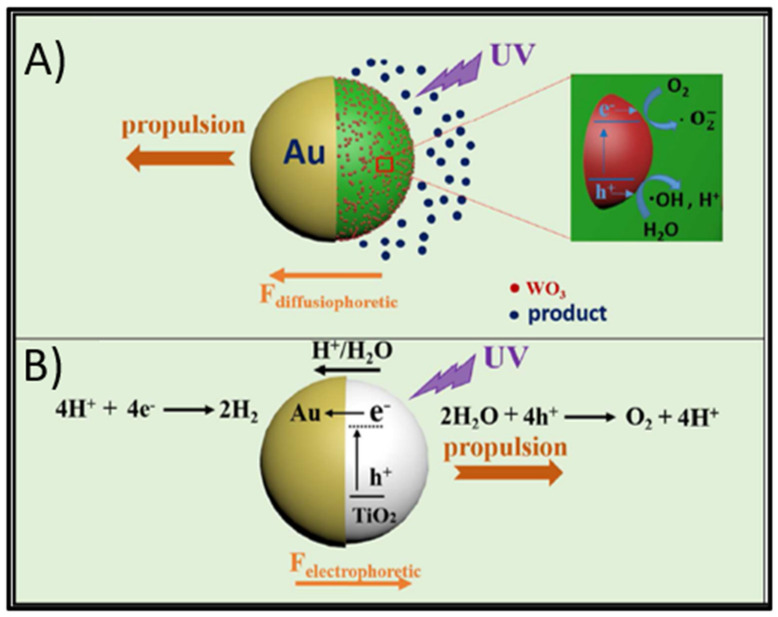
Propulsion mechanisms in light-driven semiconductor-based MNMs. (**A**) Diffusiophoresis. Asymmetrical decomposition of a fuel molecule establishes a concentration gradient, pushing the structure via diffusion of product species. Example: Au-WO_3_@C Janus micromotors. (**B**) Electrophoresis. Water redox reactions lead to an accumulation of H^+^ ions and electrons on opposite hemispheres, inducing an electric field under which a MNM can move due to its overall surface charge (usually negative). Example: Au-TiO_2_ micromotors. (**A**,**B**) Adapted with permission from Zhang et al. ACS Appl. Mater. Interfaces 2017, 9, 5, 4674–4683 Copyright 2017, American Chemical Society [61].

The first mechanism, diffusiophoresis (Figure 1A), is based on the diffusion of chemical species or ions under a concentration gradient promoted by the asymmetrical generation of product molecules along the surface of a micro/nanomotor [14,32,41,42]. The second one, electrophoresis (Figure 1B), is mostly exhibited by fuel-free Janus MNMs and is based on the influence of a self-induced electric field: one hemisphere (usually a metal layer) acts as an electron acceptor while the other (the main semiconductor) keeps the photogenerated holes; such separation allows the charge carriers to migrate to the surface of the MNM to participate in water reduction (electrons) and oxidation (holes) reactions. These redox reactions are carried out at different speeds, leading to a high concentration of H^+^ on the semiconductor surface and an accumulation of electrons on the opposite hemisphere. This charge accumulation establishes an electric field pointing towards the electron-acceptor hemisphere, which will move the MNM in a direction dependent of its overall charge (negative: semiconductor side forward; positive: electron-acceptor side forward) [12,19,35,56]. The third light-driven propulsion mechanism, bubble propulsion [64], which requires a set of conditions: (a) a fuel that can be decomposed into gas molecules (usually H_2_O_2_), and (b) a geometrical shape that promotes the formation and ejection of bubbles from an inner cavity, which can be obtained by tailoring semiconductor materials into nanotubes with variable inner diameter [32]. As fuel molecules are decomposed on the semiconductor surface, the confined space in the inner part of the tubes promotes the accumulation, growth and ejection of bubbles through one opening, pushing the micromotor in the opposite direction [32,58,64]. 

The asymmetry required to enable diffusio- and electrophoretic propulsion can be achieved on isotropic structures by taking advantage of the limited penetration depth of light in semiconductor materials, leading to a negative phototaxis motion mode [46,52,65]. However, better direction control and motion speeds can be obtained when chemical and/or geometrical asymmetry are introduced via: (a) the deposition of catalytic metals [12,20,35,37,38,49,61] or other semiconductors [14,48,56,62,66], (b) surface modification with functional groups [66,67], (c) controlled growth of different crystalline phases [33,42], and (d) tailoring into different shapes like chevron structures [14], bowls [37], twinned rods [49], brushes/trees [50,66], ‘‘peanuts’’ [54], coils [55,63], and wires [62]. On the other hand, as previously stated, the bubble propulsion mechanism has mostly been reported to power tubular micromotors [32,58,59], although it has also been confirmed to work for other geometries like ‘‘hollow-dumbbells’’ [67] and spheres [68].

Now that propulsion mechanisms have been thoroughly explored, it is important to understand how one can achieve precise control over the direction in which semiconductor-based MNMs will move. It has been shown that this type of micromotors can alternate between different motion modes and directions by changing the angle or the intensity of incident light [49,51] as well as exhibit flock behaviors with the capacity for cargo-transport and group reconfiguration in response to light of different wavelengths [12,35,39,48,52,53,69]. However, it is possible to obtain better control by combining light-response with sensitivity towards other external forces, like magnetic or acoustic fields [20,37,43,54,56,61,68]. Magnetic steering is normally integrated into semiconductor-based MNMs by the deposition of a layer of a magnetic material between the semiconductor and the electron-acceptor layer (like Co [20], Ni [43,61], and CoNi [56]). MNMs made with Fe_2_O_3_ can also be steered by weak magnetic fields [54,68] and even nanoscaled tracks in a textured substrate [54]. On the other hand, acoustic steering can be achieved over Au-TiO_2_ microbowl motors. These structures are capable of propelling under an acoustic field and accelerating or braking/reversing its motion when irradiated with UV light, a behavior that can be extended to the expansion and contraction of ensembles of microbowls [37].

As it has been reviewed in this section, the photocatalytic activity of semiconductor-based MNMs makes them perfectly suitable for the decomposition of organic pollutants, and their versatile motion dynamics can find great use for cargo-transport and drug-delivery in biomedical and environmental applications. Although some progress has been made towards the design of efficient MNMs that can work with biocompatible fuels [33,41,44,47,58] in fuel-free conditions [19,36,54] or with low energy light, new alternatives to extend the use of semiconductor-based MNMs can and should be explored.

## 3. Physical Properties of Plasmonic NPs and Hot Electrons Generation

**Figure 2:**
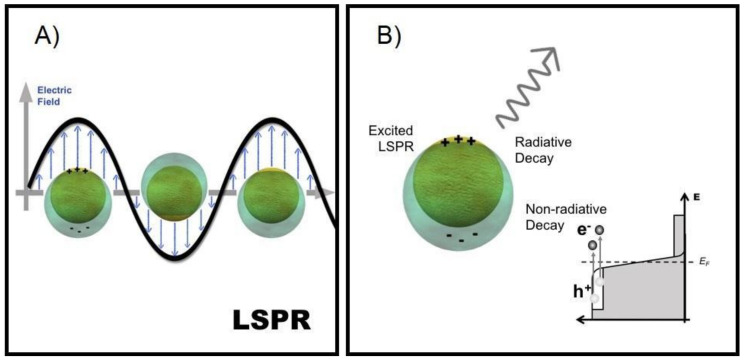
(**A**) Schematic representation of the Localized Surface Plasmon Resonance condition in Plasmonic NPs; (**B**) Once excited the LSPRs of Plasmonic NPs, it will decay either radiatively through the re-emission of a photon or non-radiatively by the generation of hot electron-hole pairs in the NPs.

Noble metal NPs have attracted great attention due to their fascinating physical and chemical properties, prevailing over their optical characteristics. The interaction of a beam of light with metal NPs makes their free electrons to be driven by the alternating electric field from the incident light. For metal NPs with a size much smaller than the wavelength of incident light, a polarization of surface charges is generated when they are excited by light (Figure 2A). When the natural frequency of collective oscillation of free electrons against the restoring force of positive nuclei matches the frequency of the incident light, there is a resonance condition known as Localized Surface Plasmon Resonance (LSPR) [70,71,72,73,74]. 

This LSPR condition enables an efficient transfer of electromagnetic energy from the far-field to the near-field of metal NPs and vice versa, a characteristic that makes them be considered as nanoantennas [71]. The LSPR of metal NPs results in the intense absorption of light and the enhancement of the local electromagnetic field near the surface of the metal NPs. Therefore, this physical process allows the Plasmonic NPs to collect the energy of visible light and concentrate it near the NPs surface [72].

The LSPR property of metal NPs can be determined by several factors, such as the size, shape, and composition of the Plasmonic NPs, together with the dielectric properties of the surrounding medium. Then, by modifying such parameters, it is possible to design NPs that interact with the entire solar spectrum [73,74].

Nowadays, high quality Plasmonic NPs can be prepared by colloidal chemical methods in large quantities with the desired physicochemical properties without the need of specialized equipment [70,75]. The versatility of the colloidal preparation methods is based on two steps: (i) the reduction of metal ions to form small metal nuclei (seeds); and (ii) the growth of those tiny nuclei into large NPs with well-defined size and morphology [70]. Complicated morphologies with high uniformity can be prepared such as: nanoplates, nanospheres, octahedra, nanoframes, nanocages, and even NPs with high Miller index facets like hexoctahedra or concave nanocubes [70,75]. 

**Figure 3:**
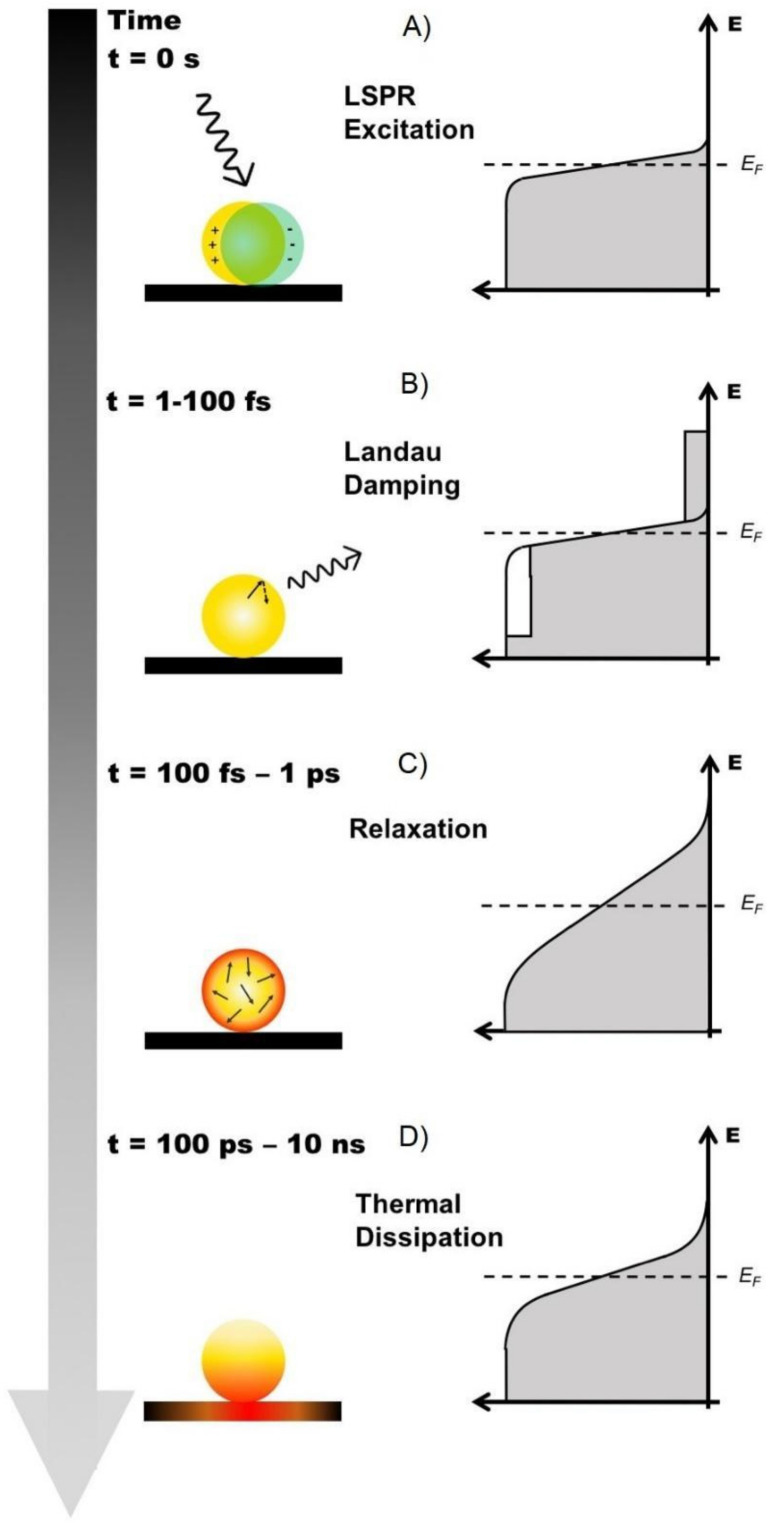
(**A**) Once the LSPR has been excited in the Plasmonic NP, the energy stored in the elevated LSPR fields is dissipated within a very short time period. Non-radiative absorption results in the generation of energetic charge-carriers in the metal NP which follow a cascade of complex processes originated after the illumination of a metallic NP, such as: (**B**) Landau Damping, (**C**) Relaxation, to end up with (**D**) Thermal Dissipation with the surrounding medium.

Plasmonic NPs can perform charge-carrier mediated reactions under visible light illumination. This is of particular importance when propulsion routes to achieve movement are pursued for MNMs. The energy stored in the elevated LSPR fields is dissipated either through radiative photon scattering or non-radiative absorption in the metal NPs within a very short time period (Figure 2B). Non-radiative absorption results in the generation of these energetic charge-carriers in the metal NPs [31,72,76,77,78]. These charge-carriers follow a cascade of complex processes originated after the illumination of a metallic NP [31,72,76,77]. Therefore, the first step in the excitation of a charge-carrier is the absorption of a photon (Figure 3A), which can be enhanced by exciting LSPR in Plasmonic NPs. Then, the LSPRs in NPs can be damped radiatively by re-emission of a photon or non-radiatively through the creation of a hot-electron hot-hole pair via Landau damping (Figure 3B) [72,76]. Since the work function of conventional Plasmonic metals are larger than their LSPR energies (E_LSPR_), hot electrons will have negative energies ranging from the Fermi level (E_F_) up to E_F_ + E_LSPR_ and cannot escape into vacuum. In the first 1–100 fs following Landau damping, the athermal distribution of the hot electron-hole pairs decay either through the re-emission of photons or through carrier multiplication caused by electron-electron interactions [76]. Between the next 100 fs to 1 ps, a Fermi-Dirac like distribution will be formed due to the redistribution of hot carriers’ energy by electron-electron scattering processes (Figure 3C). The reduced velocity of these lower energy electrons allows them to interact with phonons, then, the subsequent equilibration with the lattice occurs over a longer timescale of several picoseconds. Finally, heat is dissipated with the surrounding of the metallic NPs within 100 ps to 10 ns timescale (Figure 3D) [72,76].

**Figure 4:**
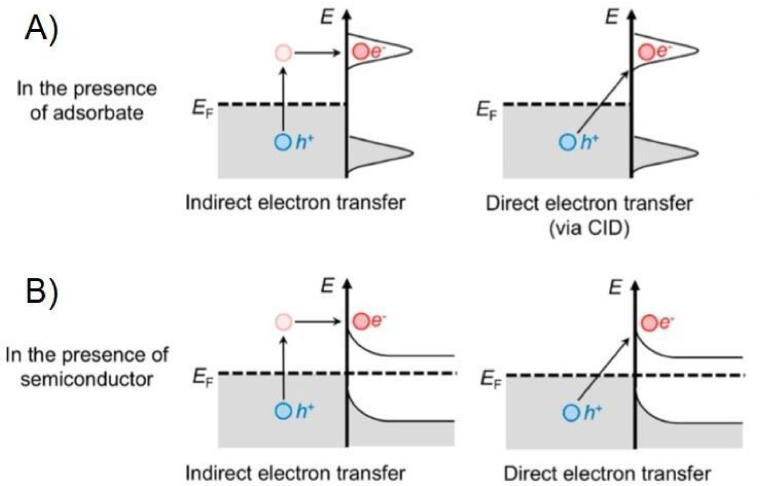
(**A**) When there is presence of adsorbate molecules on plasmonic NPs, the charge-carrier generation process through the LSPR decay can follow two main mechanisms of electron transfer into acceptor adsorbate molecules as depicted. (**B**) Similarly, the presence of a semiconductor material in contact with the plasmonic NPs will cause an interaction during the charge-carrier generation through the LSPR decay transferring hot electrons into the neighboring material as illustrated. (**A**,**B**) Adapted with permission from Zhang et al. Chem. Rev. 2018, 118, 6, 2927–2954, Copyright 2018, American Chemical Society [77].

These generated charge-carriers are considered hot since their energies are larger than those of thermal excitations at ambient temperature. Such hot-electrons can be collected to facilitate chemical reactions by being transferred into unoccupied levels of acceptor molecules on the nearby of Plasmonic NPs inducing photochemical transformations [76,77,79]. The two main mechanisms of LSPR-mediated electron transfer into acceptor adsorbate molecules correspond to indirect electron transfer or to direct electron transfer (Figure 4A) [72,74,77]. For the indirect electron transfer, the electrons are transferred into unoccupied levels of the adsorbate molecule after hot carrier generation [72,77]. Meanwhile, for the direct electron transfer LSPR, decay results in direct electronic excitation at the adsorbate-metal interface [72], also referred as chemical interface damping (CID), which induces the direct generation of hot electrons in the electron-accepting orbitals of the adsorbate [9,10]. The energized charge-carriers transiently populating otherwise unpopulated orbitals centered on the adsorbate molecule can form transient negative ions (TNI). In this process, the entire adsorbate-NP system is in an excited state where the activation of chemical bonds and chemical transformations can occur [74]. These routes concentrate and channel the energy of visible light into adsorbed molecules, enhancing the rates of chemical transformations [78].

Nevertheless, the strong metal-adsorbate interaction required for surface orbital hybridization is not easily achieved for some reactions on Plasmonic NPs. It is known that Au is chemically inert in various environments, which makes a challenge the formation of strong molecular attachments for certain reactions [77]. An alternative option is the formation of hybrid arrays with contact between Plasmonic NPs and semiconductor materials. Plasmon mediated electron transfer pathways can effectively prolong the lifetime of hot-electrons transferred to the conduction band of the semiconductors and therefore makes them capable of fostering various surface chemical reactions [77].

Therefore, plasmonic energy conversion has been proposed as an alternative to conventional electron-hole separation in semiconductor devices [31]. As described above, LSPR in Au and Ag NPs can transfer energy to hot-electrons, and an efficient mechanism for capturing such hot-electrons is to form a Schottky barrier between the metallic NP and an appropriate semiconductor [31]. TiO_2_ is a good electron-accepting metal oxide due to the high density of states in its conduction band, however, its large bandgap (~3.2 eV) limits its photo-absorption to only the UV-region of the solar spectrum (~5%). The energy needed for the hot-electron from Plasmonic NPs to overcome the Schottky energy barrier in the hybrid system is considerably smaller than the bandgap of the semiconductor, making possible to harness the energy from visible light photons by the presence of Plasmonic NPs. Moreover, the Schottky energy barrier at the metal-semiconductor interface assists in the trapping of the hot-electrons transferred to the conduction band of the semiconductor by delaying them from traveling back to the metal NP, giving them a longer lifetime to foster surface reactions on the semiconductor [77]. Again, there are two main electron-transferring routes for the charge injection from Plasmonic NP to the semiconductor material: indirect electron-transfer or direct electron-transfer (Figure 4B). As depicted in Figure 4B, the indirect electron-transfer occurs to the semiconductor conduction band after hot carrier generation, while chemical interface damping can induce the direct generation of hot-electrons in the electron-accepting conduction band of the semiconductor [77]. It should be taken into account, that after injection of hot-electrons into the neighboring semiconductor, the Plasmonic NPs are left positively charged because of the electronic depletion, which will need of an electron-donor solution for the holes scavenging [31]. 

There is one more energy-transfer mechanism for these hybrid Plasmonic NPs-semiconductor material systems not described yet. It corresponds to the interaction of the semiconductor with the strong LSPR induced EM field localized nearby at the Plasmonic NPs. As the rate of electron-hole pair formation in a semiconductor is proportional to the local intensity of the electric field, the rate of electron-hole pair formation can be considerably enhanced by this strong electromagnetic near-field enhancement nearby the Plasmonic NPs upon LSPR excitation [73]. Then, optimizing the design of plasmonic NPs into morphologies to maximize the local electromagnetic field should have a positive impact.

Finally, it is worth mentioning that the LSPR excitation and its subsequent non-radiative decay as a potential source of heat, since as described above (see Figure 3D), this non-radiative decay process ends up with a thermal dissipation with the surrounding medium inducing temperature increments at the Plasmonic NPs surface. This light-induced thermal heating of Plasmonic NPs can also play an important role to induce the motion of MNMs.

It should be remarked then, that the LSPR excitation in Plasmonic NPs can serve to generate hot electron-hole pairs at the NPs to drive chemical reactions, the subsequent heat dissipation of the NPs with the surrounding medium, and an enhancement of the electric near-field at the NPs to induce photo-catalytic reactions rate increment. These three phenomena can be harnessed for the propulsion of MNMs driven by light.

## 4. Plasmonic Photocatalysts for Light-Driven Reactions

The main challenge to obtain the desired products from a chemical reaction is to fulfill the specific conditions required to make the reaction thermodynamically favorable. This includes, but it is not limited, to provide heat to the reactants, a specific pressure, a supply of electrons or holes, as well as the presence of a catalyst. Nanoparticles have long been used in heterogeneous catalysis, for example, in catalytic converters [80]. In this section, we will provide recent examples on how plasmonic NPs can be used for light-driven reactions. In other words, the inclusion of light as an element to improve or promote chemical reactions with plasmonic NPs as the catalyst.

### 4.1. Plasmon Induced Reaction by Charge Carriers

Once the LSPR of plasmonic NPs has been excited, one of the two possible decay routes (non-radiative) will generate electrons (called hot-electrons) with more energy than the Fermi level and lower than the vacuum energy with their respective couple (called hot-holes) (see Figure 2B). The highly energetic charge carriers can be transferred to adsorbates producing the activation of chemical bonds and chemical transformations. 

**Figure 5:**
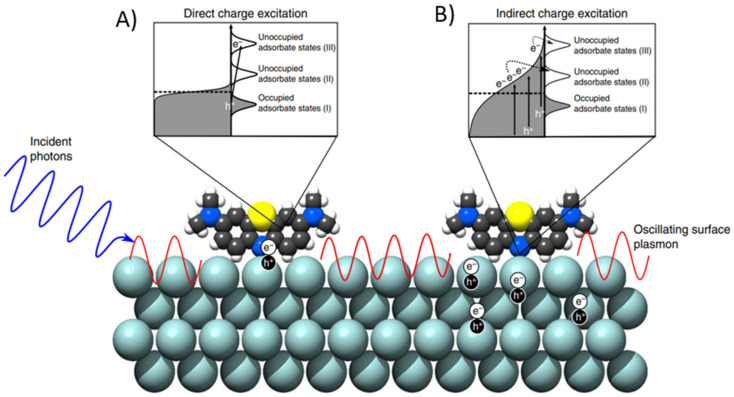
As a result of the plasmon decay, the formation of charge carriers can produce a direct or an indirect charge transfer. (**A**) The direct transfer allows the excited electron to directly occupy an empty higher energy level. (**B**) Indirect transfer requires the formation of an electronic distribution, then an excited electron can be transferred into an absorbate orbital. Adapted with permission from Boerigter et al. Nat. Commun. 2016, 7, 10545, Copyright 2016, SpringerNature [81].

In order to achieve the charge transfer from the LSPR decay into an adsorbate molecule on the plasmonic NP, two mechanisms have been proposed: the indirect and direct charge-transfer mechanisms by (see Figure 5) [74]. In the indirect transfer mechanism, an electron distribution with a high concentration of low-energy carriers within the metal NP is assumed. Then these excited electrons can fill lower-energy unoccupied states in the absorbate. On the other hand, the direct transfer mechanism does not require an excited electronic distribution on the NP, instead, the electrons from the nanoparticle-absorbate complex can be promoted directly to a higher-energy state. Although, the main contributor (direct or indirect transfers) in a plasmon-mediated photocatalytic reaction is still a topic of debate, some authors, like Boerigter et al., have provided evidence of direct charge excitation as the dominant mechanism by studying the effect of Ag nanocubes and methylene blue under laser illumination. 

**Figure 6:**
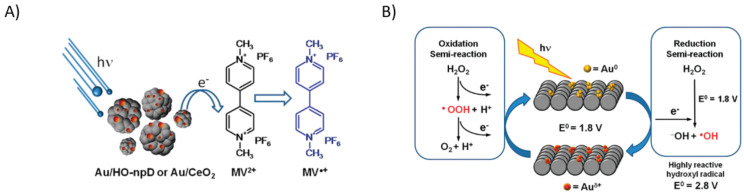
(**A**) Charge transfer of the hot-electrons to a probe molecule (MV^2+^). Adapted with permission from Navalon et al. J. Am. Chem. Soc. 2011, 133, 7, 2218–2226. Copyright 2011, American Chemical Society [82]. (**B**) Hot-electrons applied to make the Fenton reaction for the AOP in the phenol degradation, and it’s the closing cycle reducing the oxidized gold to gold 0. Adapted with permission from Navalon et al. J. Am. Chem. Soc. 2011, 133, 7, 2218–2226. Copyright 2011, American Chemical Society [82].

To gain insight into the charge injection, Navalon et al. proposed an experiment using methyl viologen (MV^2+^) as target molecule and AuNPs as photocatalyst observing the reduction from MV^2+^ to MV^•+^ [82]. Since the only photoactive material were the AuNPs it was clear evidence of the charge transfer from the electrons generated after the plasmon decay to unoccupied states of the methyl viologen (as observed in the scheme presented in Figure 6A). As they prove that the hot-electrons can be injected to adsorbates, they selected Fenton reaction, injecting hot-electrons to the unoccupied states of the hydrogen peroxide reducing it into a hydroxyl anion and a hydroxyl free radical (the generation of those Fenton reagents was observed by the degradation of phenol to catechol under 532 nm light). To regenerate the catalyst due to the gold oxidation, they let the hydrogen peroxide to be converted into oxygen gas releasing one electron and therefore reducing the oxidized gold (see scheme in Figure 6B). They presented clear evidence that the plasmonic photoactivity was due to the transfer of hot-electrons to the LUMO states of the hydrogen peroxide. 

**Figure 7:**
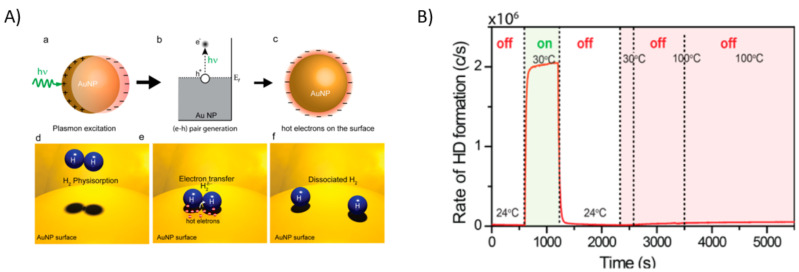
(**A**) a scheme of the localized surface plasmon resonance, (**A**) b the generation of the hot-electron above the Fermi Energy, (**A**) c the hot-electrons on the surface of the nanoparticle, (**A**) d–f schemes for the hydrogen dissociation due to the hot-electrons on the surface of the gold nanoparticle. Adapted with permission from Mukherjee et al. Nano Lett. 2013, 13, 1, 240–247. Copyright 2013, American Chemical Society [25]. (**B**) Real-time detection of the HD formation during the laser irradiation and the no thermal contribution to this reaction. Mukherjee et al. J. Am. Chem. Soc. 2014, 136, 1, 64–67 Copyright 2014, American Chemical Society [84].

Mukherjee et al. applied the idea of using hot-electrons from one AuNPs for hydrogen molecules. Interestingly, they performed the reduction to achieve a dissociation (as observed in Figure 7A) by splitting the hydrogen molecule with hot-electrons [26]. To prove the idea of hydrogen dissociation, they used hydrogen and deuterium to generate HD, observing that when the laser light was turned on, the HD formation was detected. It is also important to remark that the HD was not detected in dark conditions at high temperature (Figure 7B), which is an indication that there are no thermal contributions [83]. Demonstrating that the plasmonic photocatalysis can enable the hydrogen dissociation at room temperature by utilizing the hot-electrons, therefore, opening the possibility of populating specific states of other molecules by tuning the LSPR of metallic nanoparticles.

**Figure 8:**
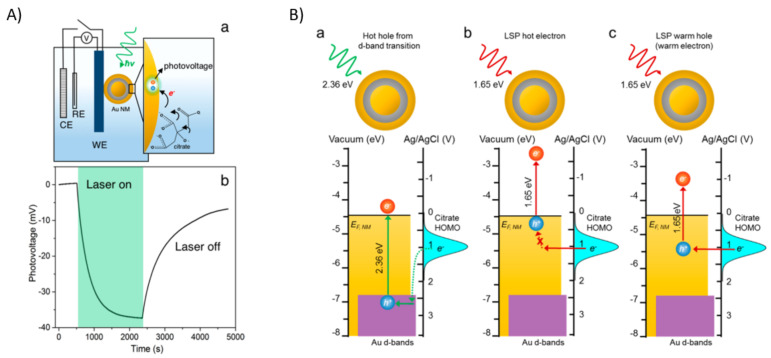
(**A**) a Scheme for the citrate oxidation under 2.36 eV of laser irradiation, and (**A**) b Open Circuit Potential measurements. Adapted with permission from Schlather et al. J. Phys. Chem. Lett. 2017, 8, 9, 2060–2067 Copyright 2017, American Chemical Society [25]. (**B**) a Scheme for the oxidation of the citrate employing the hot-electrons using the BD mode. (**B**) b Scheme where cannot be achieved the citrate oxidation in the ABD mode with warm-electrons, and (**B**) c Citrate oxidation using the ABD mode. Adapted with permission from Schlather et al. J. Phys. Chem. Lett. 2017, 8, 9, 2060–2067 Copyright 2017, American Chemical Society [25].

A core-shell gold/SiO_2_@Au NPs (nanomatryoshkas) were synthesized to study the photo oxidation induced by LSPR decay, when the NPs were irradiated with green light (exciting the LSPR) they observed under the open circuit conditions an increase in negative potential (photovoltage) which can only be explained by injection of electrons from the adsorbate to the electrode (see Figure 8Aa,Ab). The mechanism behind this behavior was explained by Schlather et al. [25] as the generation of hot-holes from the excitation of the Au d-band electrons, which were more reactive for recombining with electrons from the citrate HOMO, rather to the excitation from electrons in the gold surface (see Figure 8Ba). 

### 4.2. Plasmon Induced Heating

After the excitation of LSPR in a nanoparticle, the absorbed energy will decay and finally end up as heat. This plasmon heating effect increases dramatically the temperature around the NPs [85] and can be used to promote phase changes e.g., liquid water to water vapor without the need of heating the whole solution volume [86].

The plasmon heating effect is highly localized, according to the spot size of the laser that is used; and fast to achieve, in the picosecond time scale. Thanks to these characteristics, reactions like reforming ethanol to hydrogen or the decomposition of Dicumyl Peroxide are possible [87,88]. Moreover, these works highlighted an important aspect: it is not necessary to use high amounts of energy to produce these reactions. Therefore, the possibility in size reduction of the reactors for applications at the nanoscale.

## 5. Plasmonic NPs—TiO_2_ Hybrid Nanostructures for Light Driven Reactions

Plasmonic NPs can be mixed with TiO_2_ materials to obtain hybrid nanostructures. Titanium dioxide is a robust nontoxic material with outstanding photocatalytic properties. However, as a wide band gap semiconductor (~3.2 eV) TiO_2_ can only absorb UV light, narrowing its ability to harness the solar spectrum radiation. Therefore, TiO_2_ can be used in conjunction with Plasmonic NPs, to make use of the excellent visible light absorption properties of the Plasmonic NPs. Moreover, as described in Section 3, the interface of TiO_2_–Plasmonic NPs can offer a route for capturing hot charge-carriers from the Plasmonic NPs into the TiO_2_, since titanium dioxide corresponds to a good electron-accepting metal oxide due to the high density of states in its conduction band. All these characteristics make these hybrid materials good candidates to extend the photocatalytic activity to the visible and near-IR regions for light driven reactions.

Pioneering efforts to unveil the underlying mechanism behind the enhanced photocatalytic activity of these hybrid systems were reported by Tatsuma’s group [89,90,91]. They observed significant changes in the potential and photocurrent achieved by a TiO_2_ electrode with AuNPs or AgNPs (5–20 nm diameter) when irradiated with visible light. They obtained maximum potential responses at the LSPR mode wavelength (AuNPs ~ 540 nm; AgNPs ~ 440 nm). At the time, they proposed as a possible mechanism a plasmon-induced charge separation [89]. Later, Tian and Tatsuma gathered further evidence to conclude that photoexcited electrons from AuNPs are injected into the conduction band of TiO_2_ in response to the LSPR excitation with visible light. They evaluated electron transfer from a donor (hole scavenger) to the AuNPs, proposing that irradiation with visible light generates a photoexcited state in AuNPs or AgNPs (LSPRs) which causes that photoexcited electrons are transferred to the TiO_2_ bulk, where they will participate in different reactions, while the oxidized metal NPs receive electrons from the electron donor in the electrolytic solution [90]. Subsequently, they studied the effect of NPs size (15, 40 and 100 nm in diameter) in the quantum efficiency and maximum photocurrent for the AuNPs-TiO_2_ system. Larger NPs exhibited greater quantum efficiencies, revealed by a steeper increase in photocurrent [91].

Then, Kowalska et al. reported plasmon-induced photocatalytic chemical reactions using a 2%wt Au photodeposited with UV light onto the surface of fifteen commercial TiO_2_ powders, using methanol as a sacrificial hole scavenger [92]. The activity of these photocatalysts in the photo-oxidation of 2-propanol was evaluated by chromatographic analysis of the generated acetone. The presence of AuNPs allowed all tested photocatalysts to react under visible light irradiation. The action spectra (in terms of quantum yield) of all tested Au-TiO_2_ samples resembled their respective absorption spectra (peak positions at 575–585 nm). They proposed that the LSPR excitation of AuNPs could aid for the injection of electrons into the conduction band of TiO_2_ and reduce adsorbed O_2_ on the semiconductor surface; while the electron deficient AuNPs would then oxidize organic compounds, such as 2-propanol, and go back to its metallic state [92]. Further research showed that noble metal NP-loaded TiO_2_ was able to perform other types of reactions under visible light such as splitting of water [93]. Hybrid materials containing different gold percentages were prepared with P25 TiO_2_. Photocatalytic hydrogen formation with UV and visible light in the presence of Au-TiO_2_ was addressed, using methanol or EDTA as sacrificial reducing agents, with a Xe-doped Hg lamp as UV light source. In order to prove the visible light photocatalytic activity of Au (1.5%wt)-TiO_2_ for hydrogen generation, the second harmonic of a Nd:YAG laser at 532 nm (close to AuNPs excitation wavelength, ~550 nm) was used. A comparison between the activity of this material and bare-TiO_2_ for monochromatic (λ ~ 532 nm) and polychromatic (λ > 400 nm) irradiation showed that bare-TiO_2_ presents no activity in these conditions, while there is a significant increase in the volume of generated H_2_ when AuNPs are incorporated in the semiconductor. The same set of samples was used for the study of the oxygen generation reaction. No oxygen was evolved when using only P25 TiO_2_, while the presence of AuNPs introduced visible light photoactivity [93].

The photocatalytic activity increments in the water splitting reaction under visible illumination, was described by an alternative mechanism by Liu et al. with a model based on the near-field optical enhancement of noble metal NPs near the TiO_2_ surface, rather than by the direct transfer of charges between the Plasmonic NPs and the metal oxide [94]. They performed photocatalytic water splitting and the photocurrent of anodic TiO_2_ with and without AuNPs irradiated with UV and visible light was measured [94]. Under UV illumination, the introduction of AuNPs lead to a decrease of the photocurrent; while under visible light irradiation, AuNPs lead to an increase in the photocurrent, due to the large plasmonic enhancement of the local electromagnetic fields which increase the electron-hole pair generation rate at the surface of the TiO_2_ with a relatively short exciton diffusion length [94]. Independently, Ingram and Linic presented evidence that the interaction of localized electric field of Plasmonic NPs with the neighboring semiconductor in a hybrid material for the water splitting reaction, allows for the selective formation of electron-hole pairs in the near-surface region of the semiconductor, which should facilitate the migration to the surface, where the photocatalytic transformations are performed [95]. The same water splitting reaction was explored with visible light with a plasmonic cell which functions by illuminating a dense array of aligned Au nanorods capped with TiO_2_ which collects and conducts hot electrons to an unilluminated Pt counter electrode for the hydrogen evolution. Then, the resultant positive charges in the Au nanorods function as holes and were extracted by an oxidant catalyst which evolves oxygen from water, emphasizing the crucial role of a charge carrier mediator (hole scavenger) at the interface between the AuNPs and the solution [96].

**Figure 9:**
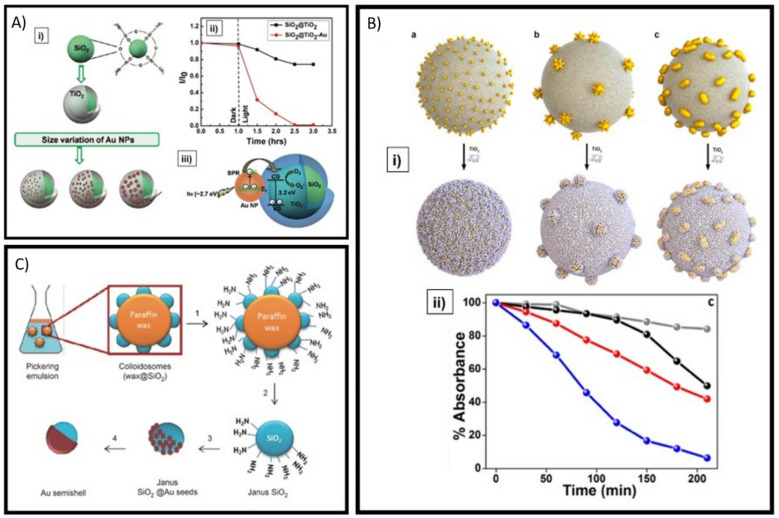
The assembly of hybrid systems incorporating plasmonic NPs-TiO_2_ presents good opportunities to develop LSPR-induced visible light active photocatalysts. (**A**) (i) The scheme shows the assembly of Silica-TiO_2_ core-shell particles decorated with AuNPs as a LSPR-induced visible light active hybrid system; (ii) presents experimental measurements of the photocatalytic activity of the hybrid system; and (iii) contains a schematic representation of the photocatalysis mechanism. Adapted with permission from Kochuveedu et al. J. Phys. Chem. C. 2012, 116, 2500–2506 Copyright 2012, American Chemical Society [97]. (**B**) (i) Anisotropic plasmonic NPs (a) nanospheres, (b) nanostars or (c) nanorods have been incorporated into the assembly of hybrid systems onto SiO_2_ particles, followed by a TiO_2_ NPs coating layer; (ii) shows experimental measurements of the photocatalytic activity of the different hybrid systems. Adapted with permission from Souza-Castillo et al. J. Phys. Chem. C. 2016, 120, 11690–11699 Copyright 2016, American Chemical Society [98]. (**C**) Colloidal methods have been reported to obtain Janus type silica particles, which can be harnessed to design novel hybrid visible light active photocatalysts as light-driven propelled micro/nanomotors. Adapted with permission from Rodriguez-Fernandez et al. Chemistry Open. 2012, 1, 90–95 Copyright 2012, WILEY-VCH Verlag GmbH & Co. KGaA, Weinheim [99].

Following all this effort, the assembly of these building blocks (Plasmonic NPs and TiO_2_ nanostructures) into strategic architectures have been evolving as well. Figure 9A.i schematizes the assembly of Silica-TiO_2_ core-shell particles decorated with AuNPs as an efficient approach to develop LSPR-induced visible light active photocatalyst hybrid systems [97]. The influence of size and density of AuNPs on the photocatalytic activity of the hybrid material were evaluated. When the photocatalytic activity of the hybrid system (SiO_2_@TiO_2_-AuNPs) was compared with the activity of SiO_2_@TiO_2_ without AuNPs under visible light illumination for the degradation of methylene blue molecules, the efficiency of the hybrid system was evident (see Figure 9A.ii). The proposed mechanism for the visible light activity is illustrated in Figure 9A.iii, where electron transfer from AuNPs to TiO_2_ during the irradiation of visible light is expected [97]. More recently, using a similar architecture, there were assembled anisotropic plasmonic NPs (nanostars, nanorods and nanospheres) onto SiO_2_ particles, followed by a TiO_2_ NPs coating layer (see Figure 9B.i) [98]. This was performed to evaluate the critical influence of the shape of Plasmonic NPs on the photocatalytic performance of the hybrid systems. The photodegradation rate of Rhodamine B was measured (Figure 9B.ii) for the three different hybrid systems (blue for Au nanostars, red for Au nanorods, black for Au nanospheres) and compared to a reference sample without AuNPs (grey). It can be seen that Au nanostars endow TiO_2_ with the strongest enhancement in photocatalytic activity, ascribed to the ability of nanostars to locally create extremely large electromagnetic field enhancements around their spikes [98]. This type of hybrid systems could be used as propelled MNMs, however it would be needed breaking the symmetry of the hybrid systems. Nonetheless, efficient colloidal approaches have been reported to obtain Janus silica particles by partly functionalizing aided by paraffin wax as schematically illustrated in Figure 9C [99]. These colloidal approaches could be extrapolated for the strategic assembly of light-driven photocatalysts hybrid systems to be used as MNMs.

**Figure 10:**
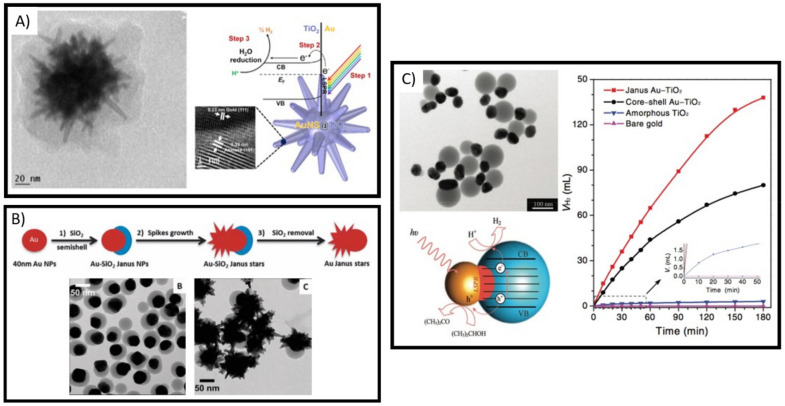
Complete hybrid Plasmonic NPs-TiO_2_ system with sizes at the nanoscale can be achieved. (**A**) Core-shell made of anisotropic plasmonic NPs coated by crystalline TiO2 layers can perform as good visible light-driven photocatalysts. Adapted with permission from Atta et al. Chem. 2018, 4, 2140–2153 Copyright 2018, Elsevier Inc. [100]. (**B**). Colloidal synthesis methods allow breaking the symmetry of plasmonic NPs and even obtaining anisotropic Janus-like NPs. Adapted with permission from Rodriguez-Fernandez et al. Chem. Commun. 2014, 50, 79–81 Copyright 2014, Royal Society of Chemistry [101]. (**C**). Non-centrosymmetric Janus Au-TiO_2_ NPs can be synthesized with excellent visible light photocatalytic activity. Adapted with permission from Seh et al. Adv. Mater. 2012, 24, 2310–2314 Copyright 2012, WILEY-VCH Verlag GmbH & Co. KGaA Weinheim [102].

The ability to implement a synthetic method to grow crystalline TiO_2_ directly on the surface of Plasmonic NPs has been a challenge hard to overcome. Recently, a sol-gel method was developed that hydrolyzes titanium precursors into crystalline TiO_2_ polymorphs at low temperature directly on AuNPs, specifically on Au nanostars, obtaining the growth of a conformal layer. This methodology enables to obtain complete hybrid Plasmonic NPs-TiO_2_ system with sizes at the nanoscale [100]. As shown in Figure 10A, TiO_2_-coated Au nanostars were obtained, which displayed significantly enhanced photocatalytic activity under visible-near IR illumination [100]. Moreover, colloidal synthesis techniques have been developed for the synthesis of Au-silica Janus nanostars as illustrated in Figure 10B, in which Au branches protrude from on half of Au-silica Janus spheres [101]. These ideas could be extended to allow the breaking of symmetry needed for a more efficient propelled movement for nanomotors. Even more, non-centrosymmetric Janus Au-TiO_2_ NPs have been achieved (Figure 10C) and tested as photocatalysts with efficient plasmon-enhanced visible-light hydrogen generation performance [102]. These advances in the precise control over the shape, size and assembly of plasmonic NPs-TiO_2_ hybrid systems would certainly motivate further advances into the light driven propelled micro/nanomotors.

## 6. Plasmon-Driven Chemical Reactions for Propelled Nanomotors

The motion design for MNMs requires three principal elements: (i) redox fuel, (ii) catalyst material, and (iii) engineering design. Here, we will focus on the plasmonic catalysts and how to select the proper fuel for each plasmonic device, for the hint to design new types of nanomachines. 

As previously described, a light-driven motor requires an asymmetric particle (Janus) to achieve the motion [1]. The chemical reaction produced by plasmon induced charge carriers can develop two possible mechanisms of motion: (i) bubble recoil, and (ii) self-diffusiophoresis.

The first motion mechanism (bubble recoil) can be performed by plasmonic NPs or plasmonic NP/Semiconductor hybrid systems that can reach the redox potentials for the water splitting reactions and overcoming the solubility limit of either hydrogen and/or oxygen, producing bubbles and therefore gaining momentum due to the detach of the bubbles. Several reports have shown the water splitting reaction by tuning the band gap to the Fermi energy of the plasmonic NPs to inject hot-electrons to the conduction band or hot-holes to the valence band for evolving hydrogen or oxygen, respectively [84,103,104].

The second motion mechanism (self-diffusiophoresis) is performed due to a reaction that only takes place in one side of the Janus motor, which increase the concentration of the specie in this side of the particle favoring an osmotic flow which finally causes a propulsion of the asymmetrical motor [27,84]. Traditionally, these kinds of motors utilize noble metals (as Au) as the inert face of the Janus motor and let the exciton produced in semiconductors to saturate the surface and perform the motion [92,93,97,98,100,102,105]. Plasmonic photochemistry has proven that the highly-energetic charge carriers can perform different types of reactions efficiently which could be used as fuel for MNMs with this motion mechanism. 

In this sense, Table 1 is a summary of the plasmonic photocatalysis reactions that can be used as a fuel for propelling micro/nano machines, for possible plasmonic light-driven MNMs.

## 7. Conclusions and Future Perspective

Currently, significant advances have been made in the mechanisms for micro/nano machines. However, there are still substantial challenges that must be overcome to apply these machines. The use of light to generate movement to the devices is strongly linked to the band gap of semiconductors. In general, they require highly energetic light, limiting their use in specific applications of medicine. In this context, we believe that plasmonic photochemistry is an alternative to this challenge since the LSPR of the plasmonic NPs can be tuned by the shape, size, and type of metal used.

The photothermic effect of plasmonic NPs has been extensively studied, but in the past years notable research has been developed in the plasmonic photochemistry field, which could be extended into the MNMs field by taking advantage of the highly energetic charge carriers in light-driven machines.

## Figures and Tables

**Table 1:** Fuels for propelling micro/nano machines.

Material	Possible Fuel	Reaction	Carrier	Wavelength	Ref.
Au@SiO_2_ Matryoshka	Citrate	Oxidation	hot-hole	2.36 eV and 1.65 eV	[27]
Au/NiOx/Al	H^+^	Reduction	hot-electron	White light	[103]
AuNPs/TiO_2_	Hydrogen	Oxidation/dissociation	hot-electron	White light	[83]
Ag-Nanocubes	Oxygen	Reduction	hot-electron	White light	[84]
Au/LaFeO_3_	Water	Oxidation	hot-hole	White light	[104]
Au/CeO_2_	H_2_O_2_	Reduction	hot-electron	2.36 eV	[82]
AuNPs@TiO_2_	Water	Oxidation	Hot-hole	>400 nm	[93]
AuNPs@TiO_2_	2-propanol	Oxidation	Hot-hole	>450 nm	[92]
Au-CoreShell Silica@TiO_2_	Methylene blue	Advance oxidation	Hot-electron	>420 nm	[97]
AuNPs/AuNRs/AuNSs@TiO_2_	Rhodamine B	Advance oxidation	Hot-electron	>350 nm	[98]
TiO_2_-coated AuNSs	Water	Reduction	Hot-electron	200–1500 nm	[100]
Au-Silica Janus NSs	Isopropanol	Oxidation	Hot-holes	>400 nm	[102]
